# Supplementary material for: Development of a scale for the evaluation of the quality of the shared decision process in multiple sclerosis patients
Source: PLoS One. 2022 May 13;17(5):e0268125. doi: 10.1371/journal.pone.0268125 (PMC9106213; doi:10.1371/journal.pone.0268125)
Supplement: Attachment — Submitted filename: Respuestas.pdf [file pone.0268125.s002.pdf]

Reviewer #1

1. **You mentioned that you used PCA to select the factor structure. Are there other clustering methods you could have used to achieve the same/similar results?**

   Yes, indeed. We could have used non metric multidimensional scaling and cluster analysis for ordinal data, for instance.

   Non metric multidimensional scaling is a statistical methodology used to visualize high dimensional and complicated data sets in few dimensions (preferably in two). The goodness of the fit is made through the stress parameter. Stress could be described as a value showing the difference between the distance in the reduced dimension in comparison to the complete dimensional space. Stress values greater than 20 are considered as a poor dimension selection, values between 10 and 20, reasonable, between 5 and 10, good, between 2.5 and 5, excellent and between 0 and 2.5, perfect.

   The principal goal of cluster analysis for ordinal data is to classify the observations in groups, in the way that, the categorical observations in one group are most similar to each other and the differences between the groups are the most different as possible. This technique can be used as dimensional reduction as it is able to describe the hidden structure of the objects.

   Nevertheless, to perform principal components analysis was for us a priority because this statistical tool is able to rank the dimensions/components based on the amount of variance explained by the data and we want to explain the maximum amount of information by the new selected dimensions.

   $$Var(PC_1) > Var(PC_2) > ... > Var(PC_N)$$

   Where N are the number of original variables and PC are the principal components.

2. **You mentioned you used Leave-One Item out Cross-validation (i.e. you deleted one item out, and re-examine their alpha value), which may be biased as the model was trained/built using the entire data. To avoid this kind of bias, one will have to do a Leave-one-out cross-validation (Leave one person out of the model) and refit the model with that person included, and check whether or not the alpha value changes. If the change in the alpha value with and without this person is too drastic, then the model is unstable with respect to the selected items. In that case, one will need to redefine the factor structure or use limited factors. This is what I call model validation. Therefore, in addition to the leave-one item out approach, that evluates the consistency of the factor structure, I will like the authors to do leave-one out validation (leave-one person out of the model) and refit their model with the 4 identified scales. You can then plot the observed alpha versus the predicted alpha to see how well the model agrees. This will guarantee that an already validated scale is applicable to Spanish cohort. Unless otherwise, please provide concrete details why this sort of analysis cannot be done. I am statisfied with the current results, and the achieve value of alpha =0.88.**

   Cronbach's alpha coefficients using the whole sample N = 203 are presented in the table 1.

Table 1: Reliability analysis

	raw_alpha	std.alpha	G6(smc)	average_r	S/N	alpha se	var.r	med.r
Item01	0.88	0.88	0.93	0.23	7.36	0.01	0.03	0.18
Item02	0.87	0.88	0.93	0.23	7.22	0.01	0.03	0.18
Item03	0.87	0.88	0.93	0.23	7.11	0.01	0.03	0.18
Item04	0.87	0.88	0.93	0.23	7.09	0.01	0.03	0.18
Item05	0.87	0.88	0.93	0.23	7.19	0.01	0.03	0.18
Item06	0.87	0.88	0.93	0.23	7.14	0.01	0.03	0.18
Item07	0.88	0.88	0.93	0.23	7.34	0.01	0.03	0.18
Item08	0.87	0.88	0.93	0.23	7.20	0.01	0.03	0.18
Item09	0.88	0.89	0.94	0.25	7.83	0.01	0.03	0.20
Item10	0.88	0.88	0.93	0.24	7.54	0.01	0.04	0.19
Item11	0.87	0.88	0.93	0.23	7.21	0.01	0.03	0.18
Item12	0.87	0.88	0.93	0.23	7.24	0.01	0.03	0.18
Item13	0.88	0.88	0.93	0.24	7.46	0.01	0.03	0.19
Item14	0.88	0.88	0.93	0.23	7.34	0.01	0.03	0.19
Item15	0.88	0.88	0.93	0.23	7.36	0.01	0.03	0.19
Item16	0.87	0.88	0.93	0.23	7.28	0.01	0.03	0.19
Item17	0.88	0.88	0.93	0.24	7.39	0.01	0.03	0.19
Item18	0.88	0.88	0.93	0.23	7.35	0.01	0.03	0.19
Item19	0.88	0.88	0.93	0.24	7.45	0.01	0.03	0.19
Item20	0.88	0.88	0.93	0.24	7.47	0.01	0.04	0.19
Item21	0.88	0.88	0.93	0.24	7.47	0.01	0.03	0.19
Item22	0.87	0.88	0.93	0.23	7.19	0.01	0.03	0.18
Item23	0.87	0.88	0.93	0.23	7.12	0.01	0.03	0.18
Item24	0.87	0.88	0.93	0.23	7.09	0.01	0.03	0.18
Item25	0.87	0.88	0.93	0.23	7.06	0.01	0.04	0.18

Where raw_alpha is the alpha that we would obtain if we deleted the item itself. (Leave-One Item out Cross-validation). The global value of raw alpha is 0.88.

And for each factor, using the whole sample:

- SatisfactionSDM
  The overall Cronbach's alpha is 0.874.

	raw_alpha	std.alpha	G6(smc)	average_r	S/N	alpha se	var.r	med.r
Item01	0.87	0.87	0.87	0.45	6.64	0.01	0.01	0.44
Item02	0.85	0.85	0.86	0.42	5.86	0.01	0.01	0.41
Item03	0.85	0.85	0.85	0.42	5.81	0.02	0.01	0.41
Item04	0.85	0.85	0.85	0.42	5.82	0.02	0.01	0.41
Item05	0.86	0.86	0.86	0.43	6.09	0.01	0.01	0.41
Item06	0.87	0.87	0.87	0.45	6.59	0.01	0.01	0.44
Item11	0.86	0.86	0.86	0.44	6.25	0.01	0.01	0.43
Item12	0.86	0.86	0.86	0.44	6.36	0.01	0.01	0.44
Item25	0.87	0.87	0.87	0.45	6.61	0.01	0.01	0.45

- AdverseEffectsDMT

The overall Cronbach's alpha is 0.925.

	raw_alpha	std.alpha	G6(smc)	average_r	S/N	alpha se	var.r	med.r
Item15	0.91	0.91	0.90	0.73	10.64	0.01	0.01	0.70
Item16	0.90	0.91	0.89	0.72	10.39	0.01	0.00	0.73
Item17	0.90	0.91	0.90	0.73	10.56	0.01	0.01	0.72
Item18	0.91	0.92	0.91	0.74	11.66	0.01	0.01	0.73
Item19	0.92	0.94	0.93	0.79	15.16	0.01	0.00	0.78

- InfoReliability

  The overall Cronbach's alpha is 0.873.

	raw_alpha	std.alpha	G6(smc)	average_r	S/N	alpha se	var.r	med.r
Item13	0.85	0.85	0.83	0.59	5.69	0.02	0.01	0.60
Item14	0.85	0.85	0.82	0.58	5.51	0.02	0.01	0.60
Item22	0.83	0.83	0.82	0.55	4.88	0.02	0.02	0.51
Item23	0.85	0.85	0.83	0.58	5.61	0.02	0.01	0.62
Item24	0.86	0.86	0.83	0.61	6.24	0.02	0.01	0.63

- DMTConvenience

  The overall Cronbach's alpha is 0.625.

	raw_alpha	std.alpha	G6(smc)	average_r	S/N	alpha se	var.r	med.r
Item07	0.54	0.57	0.56	0.25	1.30	0.05	0.04	0.16
Item08	0.54	0.54	0.54	0.23	1.18	0.05	0.04	0.14
Item10	0.64	0.64	0.69	0.31	1.81	0.04	0.06	0.16
Item20	0.56	0.53	0.53	0.22	1.13	0.05	0.04	0.15
Item21	0.56	0.54	0.54	0.23	1.17	0.05	0.04	0.15

If we perform leave one (person) out cross validation, the resulted alpha's for each subset (N-1) are plotted in figure 1.

Figure 1: Leave one person out cross validation for cronbach's alpha.

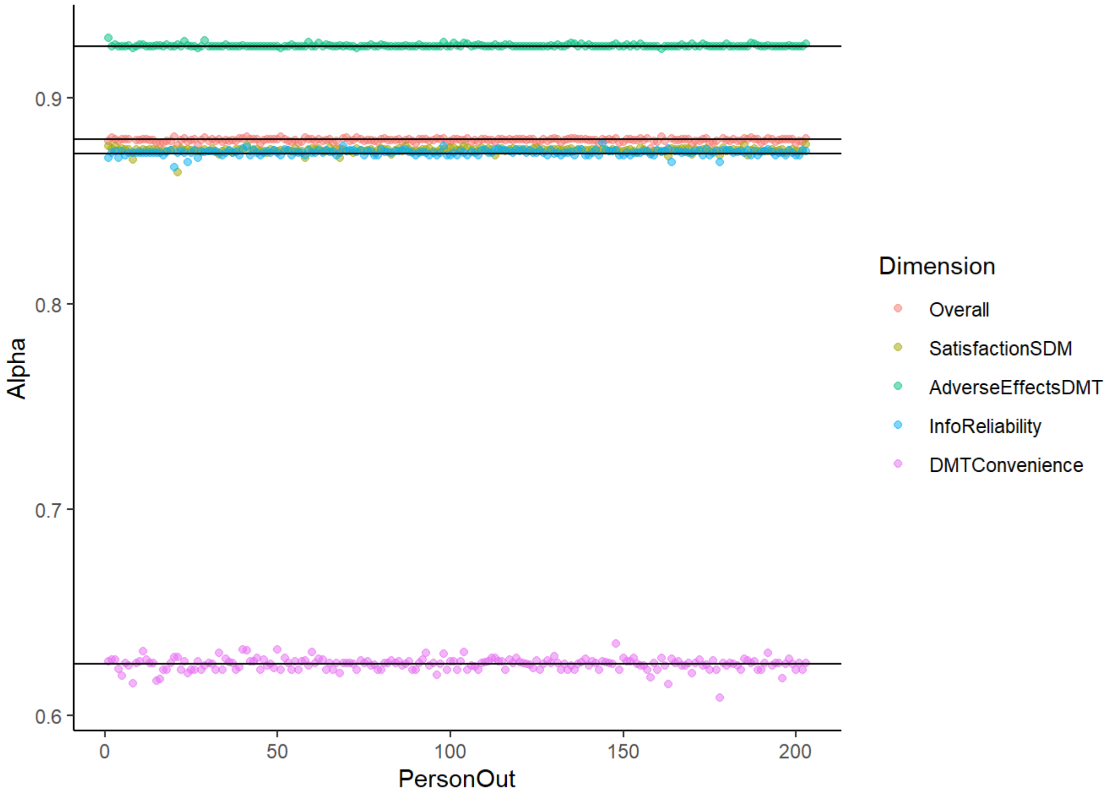

Where the solid lines are the expected alpha values and the dotted lines the observed for each dimension, as well as for the overall alpha, when all the items are included.

As we can see the observed alpha values are quite stable and they are in agreement with the expected values.

Reviewer #3 The work seems very solid to me, although the number of patients recruited is small. Only in the discussion, I think it is necessary to mention other decision-making scales validated in Spanish, such as the Control Preference Scale, and explain the advantages of using the scale evaluated in this manuscript. Other scales are now mentioned and the main differences with prior scales have been highlighted. Our scale is unique in evaluating satisfaction with the information obtained during the process itself and offers an improvement opportunity for physicians and in their relationship with patients.
